# Political embeddedness in public–private partnership for nature conservation: A land trust reserve case from China

**DOI:** 10.1007/s13280-023-01936-y

**Published:** 2023-10-11

**Authors:** Jiacheng Zhao, Tong Jin, Pei Zhang, Max Krott, Jinlong Liu

**Affiliations:** 1 Central South University, Changsha, China; 2 The Nature Conservancy, China Program, Beijing, China; 3 Humboldt University of Berlin, Büsgenweg 3, 37077 Göttingen, Germany; 4 University of Goettingen, Göttingen, Germany; 5 Renmin University of China, Beijing, China

**Keywords:** ENGO, Nature conservation, Partnership, Political embeddedness, Power

## Abstract

Private sector plays an increasingly vital role in nature conservation globally. This study explores the concept of political embeddedness, which suggests that governments and environmental nongovernmental organizations (ENGOs) can leverage each other’s strengths to achieve both formal and informal goals. Using the case of Laohegou Nature Reserve in China, this study illustrated how the complementary advantages of the government and ENGOs form the foundation of a land trust reserve. Within the case, the study found that power and interest balance between the government and ENGOs during project implementation supported their formal cooperation in nature conservation. This study proposed a political perspective to elaborate power and interest in the formal and informal dimensions of nature conservation public–private partnership (PPP) project. Moreover, it noted that a balance of power between the government and ENGOs is essential in building partnership networks with inclusive interests.

**Supplementary Information:**

The online version contains supplementary material available at 10.1007/s13280-023-01936-y.

## Introduction

Public–private partnerships (PPPs) have become an increasingly popular approach to nature conservation (Plummer and FitzGibbon 2004; Armitage et al. 2012). While direct state intervention continues to be the primary means of nature conservation, governments have sought to share responsibility with the private sector (Armitage et al. 2012; Hatchwell 2014). An increasing range of environmental nongovernmental organizations (ENGOs), corporations, communities, and research institutions have been selected as partners because of their policy influence, funding, technical expertise, and local connections (Bailey and Bryant 2005; Brechin and Salas 2011; Lambooy and Levashova 2011). In this way, the public and private sectors can leverage their respective advantages to achieve the common goal of nature conservation (Brinkerhoff 2002; Brechin and Salas 2011).

In addition to ecotourism (Romero-Brito et al. 2016), carbon markets (Lambooy and Levashova 2011), and policy initiatives (Bailey and Bryant 2005; Rozylowicz et al. 2017), private protected areas (PPAs) are an influential means by which the private sector can directly contribute to nature conservation (Stolton et al. 2014; Bingham et al. 2017). Stolton et al. (2014, p. 12) defined a PPA as “a protected area, as defined by IUCN, under private governance.” PPAs can increase the coverage and connectivity of reserves, mitigate government spending, enhance stakeholder participation, and strengthen ecological and socioeconomic linkages (Holmes 2013; Stolton et al. 2014; Cortés Capano et al. 2019). Among the various forms of PPAs, land trusts have garnered considerable interest from private actors, including ENGOs (Merenlender et al. 2004; Rissman et al. 2007). This approach involves voluntary and incentive-based protection of private land resources (Merenlender et al. 2004). For reserve habitat and open space, non-profit organizations are granted easements that impose restrictions on development and other activities concerning the land (Kiesecker et al. 2007; Rissman et al. 2007).

Although the positive role of PPAs in nature conservation is increasingly acknowledged (Shumba et al. 2020; Palfrey et al. 2021), owing to natural, political, and economic conditions, PPAs are unevenly distributed geographically (Stolton et al. 2014; Cortés Capano et al. 2019). In particular, these researches and practices tend to be concentrated in a handful of countries such as the US, Mexico, South Africa, and Australia (Fitzsimons 2015; Cortés Capano et al. 2019; Shumba et al. 2020). Yet, with ongoing private-sector development—especially international organizations—PPAs are becoming more widely disseminated (Stolton et al. 2014; Palfrey et al. 2021).

While departing from the framework of a zero-sum game between the state and society, this study acknowledged, nevertheless, that the state has a strong capacity for control, and ENGOs are motivated to maintain autonomy and seek legitimacy (Song et al. 2015; Yuen 2018). Accordingly, we adopted a political science perspective to analyze interactions in PPP projects. This study did not assume that the actors involved choose strategies in a problem-solving manner (Sack 2019). Instead, we aimed to verify that their partnerships are based on the respective power and interests (Benson 1975). To this end, we proposed the concept of political embeddedness and used it to analyze interactions between governments and ENGOs in the dimensions of complementarity and power. While the public and private sectors exploit their advantages based on the principle of complementarity, they also use the instruments of power to guarantee their informal interests (Krott et al. 2014; Rahman and Giessen 2017; Zhao et al. 2022).

This study aimed to test the political perspective in Chinese experience. China, a country seen as a typical case of authoritarian environmentalism (Beeson 2010), has gradually liberalized ENGO participation to improve nature conservation (Schwartz 2004; Xu and Byrne 2021). Since the establishment of the first nature reserve in 1956, state coercion has dominated Chinese nature conservation. However, with China’s implementation of the “reform and opening up,” as well as the progress of globalization, the government’s attitude toward ENGOs has become increasingly positive (Xu and Byrne 2021). In fact, ENGOs have grown considerably in China in recent years (Schwartz 2004; Xu and Byrne 2021). International ENGOs have been involved in biodiversity conservation in China since 1980, and China’s first civil-organized ENGO was established in 1994. By 2015, there were more than 7,000 ENGOs in China (MCA 2016). The relationships between the state and society in China are not merely characterized by one party suppressing the other (Tsai 2007; Teets 2018). Instead, certain local governments actively seek support from private actors, such as ENGOs, particularly in non-sensitive areas like nature conservation (Hsu et al. 2021; Zhao et al. 2023). In response, Chinese ENGOs have adopted a tactful approach, avoiding direct confrontation with the state and instead opting for a more adaptable strategy in their interactions. They have identified numerous acceptable avenues to participate in governance, including policy advocacy (Dai and Spires 2018; Teets 2018), public interest lawsuits (Xu and Byrne 2021), PPA management (Stolton et al. 2014; Li et al. 2021), and media campaigns (Yang 2005; Xu and Byrne 2021).Through these methods, they actively engage in governance without overtly challenging the government’s authority (Song et al. 2015; Teets 2018).

This study used the Laohegou Nature Reserve Project (hereafter, the Laohegou project) in Sichuan Province, China, as a case study to investigate complementarity and power strategies in the government–ENGO relationship. In 2012, the Laohegou Nature Reserve was established as the first land trust reserve in China to be promoted and managed by an ENGO (Stolton et al. 2014). Thus, as a pioneering case in China, the Laohegou project reflects not only the respective strengths of the government and ENGOs but also the friction and balance between them. By examining the Laohegou project, we aimed to explain how the political embeddedness between the government and ENGOs contributed to the PPP project. Specifically, we addressed the following research questions: “How do the government and ENGOs leverage their respective advantages to achieve mutually shared goals in the PPP project?” and “What types of power relationships do the government and ENGOs construct to protect their informal interests and support the PPP project?” Based on the empirical evidence, this study examined the theoretical causal mechanism in the case. In addition to contributing to research on ENGOs’ participation in China (Zhao 2004; Dai and Spires 2018), this study presents a more general analytical framework to examine interactions between governments and ENGOs.

## Theoretical background

This study developed the concept of political embeddedness and distinguished formal and informal dimensions in PPPs. The formal level concerns complementarity-based cooperation among actors (Evans 1996; Brinkerhoff 2002); the latter describes the arena where actors focus on their power and interests (Benson 1975; Sack 2019), and interactions in the informal level support the balances and partnerships among organizations (Benson 1975). While we used the theory of political embeddedness as a shorthand term to describe key variables influencing PPPs, it is still necessary to seek an instrument to observe the power and interests. Hence, this paper further adopted actor-centered power (ACP) theory which provides a theoretical framework and observation approach (Krott et al. 2014; Zhao et al. 2022).

### Political embeddedness in public–private partnerships

Organizations tend to be embedded in various interorganizational networks (Evans 1995; Gulati and Gargiulo 1999). In a PPP project, organizations have repeated interactions, coordinate policy objectives, and determine policy outputs (Brinkerhoff 2002). From the perspective of political embeddedness, the political environment, political institutions, and power structures can shape the behavior of organizations (Zukin and DiMaggio 1990; Sun et al. 2010). In the case of China, while maintaining a clear advantage, the government has established a flexible and negotiable space for ENGOs (Ho 2001; Spires 2011). To influence policy formulation and implementation, nongovernmental organizations (NGOs) in China discreetly adopt strategies to establish political connections with the government and retain autonomy (Zhao 2004; Song et al. 2015). Researchers have noted that NGO leaders maintain personal connections with government officials to influence policy making (Song et al. 2015; Teets 2018). In addition to the individual level, NGOs are more or less embedded in the Chinese bureaucracy at the organizational level, and try to ensure their legitimacy and impact on policies (Dai and Spires 2018; Yuen 2018).

By drawing on Lasswell’s (2018) “who gets what, when, and how” perspective, this study further developed the concept of political embeddedness. While power structures influence organizational strategies (Benson 1975), organizations also seek to maximize their power through their behavior (Zukin and DiMaggio 1990; Peters 2018). Therefore, we divided political embedding into formal and informal dimensions. At formal dimension, the partnership is a dynamic relationship among diverse actors intended to achieve mutually agreed-upon objectives (Brinkerhoff 2002). The complementary advantages of actors form the rational division of labor, which is the basis of PPP projects (Evans 1996; Brinkerhoff 2002; Plummer and FitzGibbon 2004).

When informal dimension is discussed, the state and ENGOs utilize their power resources to pursue for their self-interest (Krott 2005). Studies have noted that bureaucratic organizations have informal interests in terms of power, staff, and budget (Peters 2018; Zhao et al. 2022). In addition, NGOs have an informal interest in developing and implementing projects (Burns et al. 2017; Rahman and Giessen 2017), for instance, the World Bank spreads neoliberalism in the implementation of aid policies (Rahman and Giessen 2017). The complementary resources that the actors invest in the PPP contribute to the interdependence of each other. At the informal dimension, the other’s dependence on oneself becomes an element of power for the actors, through which they can realize informal interests (Avelino and Wittmayer 2016; Giessen et al. 2016).

### Actors, interest, and power

Actors are “entities that have the possibility of influencing processes in order to achieve their own goals (Schusser et al. 2015, p. 93).” Actors are driven by their interests in policy formulation and implementation (Krott 2005; Zhao et al. 2022). Interests, meanwhile, “are based on action orientation, adhered to by individuals or groups, and they designate the benefits the individual or group can receive from a certain object, such as a forest (Krott 2005, p. 8).” We adopted a theoretical definition of interest to detect the formal objectives and informal interests of actors (Zhao et al. 2022). Formal objectives are “normative expectations addressed to the occupants of given positions (Scharpf 2018, p. 64).” They are declared by individuals and organizations in both the social and ecological dimensions (Zhao et al. 2022). Informal interests are related to rational self-interested behavior linked to the self-preservation, autonomy, and growth of actors (Scharpf 2018). Informal interests involve political, economic, and strategic aspects (Zhao et al. 2022). As shown in Table 1, researchers infer the informal interests of actors by observing their behavior and benefits in relation to policy impact (Zhao et al. 2020, 2022). Political interest is the desire of actors to maximize their control over the project (Gippner 2020; Khan and Giessen 2021). Economic interest is their need to maximize financial gain (Peters 2018). Strategic interests refer to the desire of actors to preserve and export their ideologies and policy ideas (Rahman and Giessen 2017; Peters 2018).

**Table 1:** Types of informal benefits in policy impacts

Types of informal benefits	Observable facts
Control	The increase in one’s control in policy formulation and implementation
	The extension of one’s influence over external actors by using policies
Economic benefit	The increase in one’s economic gains in policy formulation and implementation
	The increase in one’s economic benefits from external actors by using policies
Dissemination of ideas	Spreading or defending one’s ideas in policy formulation and implementation
	Disseminating one’s ideas to external actors by using policies

*Source* Adapted from (Zhao et al. 2022)

Krott et al. (2014, p. 37) associated power and actors and defined power as “a social relationship in which actor A alters the behavior of actor B without recognizing B’s will.” In ACP theory, power can be measured by the resources available to the actor (Krott et al. 2014). Resources can be directly adopted by subordinates or form a basis for them to threaten others (Krott et al. 2014; Zhao et al. 2020). Such instruments are called power elements, and they encompass coercion, incentives, and dominant information (Krott et al. 2014). As Table 2 reveals, power elements can be linked to observable facts. Thus, ACP theory can provide an analytical tool to examine the sources of power and the power instruments of particular actors (Krott et al. 2014; Giessen et al. 2016).

**Table 2:** Definitions of power elements and observable facts

Power element	Definition	Observable facts
Coercion	Altering behavior by force	Physical action, threats of physical action, or sources for physical action
(Dis)incentives	Altering behavior by (dis)advantage	Providing, or threatening with, sources of material or immaterial benefit or impairment
Dominant information	Altering behavior by means of unverified information	Providing, or threating with, sources of unverified information

*Source* Adapted from Krott et al. (2014)

**Fig. 1:**
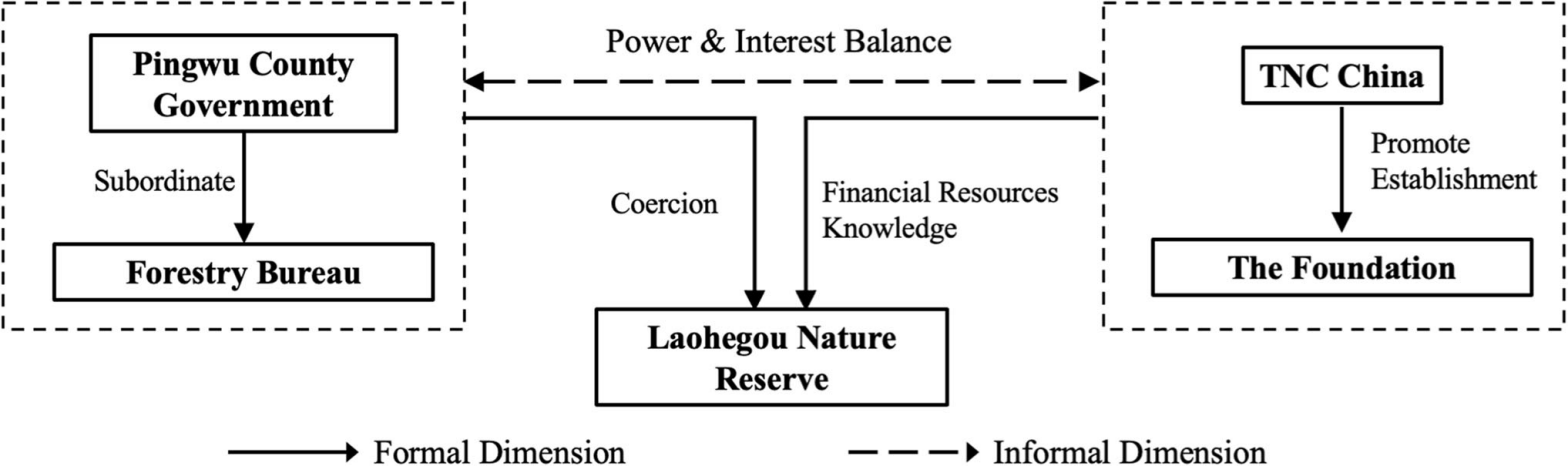
Theoretical analysis of the case

Figure 1 illustrates how we linked the theoretical background and the case of the Laohegou project. Based on the theoretical concept of political embeddedness and ACP theory, we proposed the causal mechanism in the case and developed two working hypotheses. We also kept the working hypotheses in line with the empirical evidence.

Working hypothesis 1: Based on consistent objectives, government coercion whereas monetary and informational resources from ENGOs together produce the innovative management of the Laohegou Nature Reserve.

Working hypothesis 1 suggests that the government and ENGOs have found ways to collaborate in the Laohegou project. Their formal objectives drive the partnership. Moreover, both parties use their complementary power resources to contribute to the shared expectations of nature conservation.

Working hypothesis 2: The balance of power and interests between the government and ENGOs determines support for the operation and management of the Laohegou Project.

Working hypothesis 2 suggests that PPPs can only persist if both parties maintain a balance of power. If one party is too strong, the other side’s interests, especially their informal interests, will be compromised. It could either undermine the autonomy of the subordinate, or cause it to withdraw resources. Hence, a balance of power will ensure a certain level of interest for each party. A state of relative satisfaction on both sides will support cooperation and provide a basis for innovative management.

## Materials and methods

**Fig. 2:**
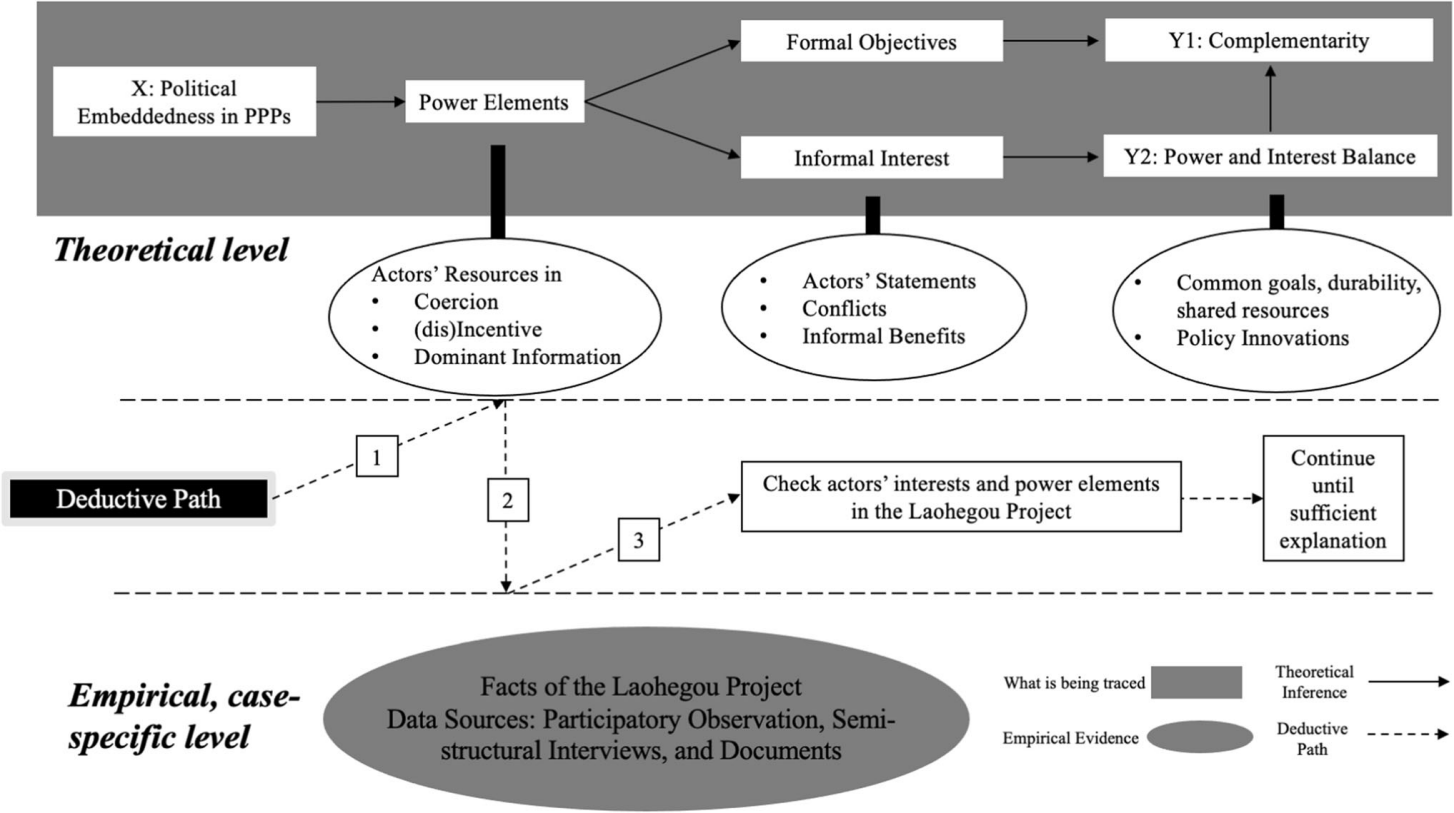
Process tracing in the case of Laohegou Project

A single case study approach is suitable to expand and generalize a theory (Yin 2018). Hence, through a structured analysis of the case material, we sought to complement the understanding of the causal mechanisms between power and cooperation in the PPP literature. Based on the case of the Laohegou project, we established a more general theory of political embeddedness. To this end, we adopted a case-centric process-tracing approach to describe a generalizable causal mechanism (Beach and Pedersen 2019). As Fig. 2 shows, we looked for observable manifestations regarding power, interests, and program effects in the empirical data. Next, we continuously checked the power and interests of the government and ENGOs and describes the causal relationships between these variables and project operations until the explanations are sufficient.

We used triangulation in the data collection and analysis to overcome bias (Flick 2022). Such bias has to do with the different perspectives of researchers with regard to answering the research questions (Flick 2022). We verified our findings using three sources of data: interviews, documents, and participant observation.

Our first method of data collection was participatory observation. Two Chinese researchers conducted field research in Pingwu County, Sichuan Province, in 2017 and revisited the area in 2018 and 2019. The researchers mainly observed the daily work of the Laohegou Nature Conservation Center (LNCC) in terms of controlling access, conducting patrols, and maintaining infrared cameras. In addition, the researcher observed the community’s involvement in nature conservation, including planting eco-friendly agriculture productions and organizing patrols for nature conservation.

Second, the primary data source was semi-structured interviews. Using background knowledge gained from the 2017 field research, the researchers first identified three government and ENGO staff involved in the Laohegou project for interviews. Based on their suggestions, additional interviewees were identified; this process is known as the snowball sampling method (Noy 2008). As Table 3 indicates, 16 interviews were conducted with the staff of international ENGO named T (hereafter, ENGO-T), Paradise Foundation staff, local government officials, LNCC employees, and members of local communities. As the Appendix A shows, the interview content involved “the process of establishing the Laohegou project,” “funding sources,” “personnel management,” “the effect of nature conservation,” “community development,” and “challenges in project operations.”

**Table 3:** Sources of interview data

No	Affiliation	Date	Place
1	Government official	2018.08.22	Mianyang
2	ENGO staff	2018.08.28	Pingwu
3	ENGO staff	2018.08.30	Pingwu
4	Government official	2018.09.01	Pingwu
5	Government official	2018.09.03	Pingwu
6	Government official	2018.09.04	Pingwu
7	Government official	2018.09.10	Pingwu
8	ENGO staff	2018.9.16	Chengdu
9	ENGO staff	2018.9.21	Beijing
10	Member of the local community	2019.08.25	Pingwu
11	Member of the local community	2019.08.25	Pingwu
12	Member of the local community	2019.08.26	Pingwu
13	Member of the local community	2019.08.27	Pingwu
14	ENGO staff	2020.05.13	Online interview
15	ENGO staff	2022.03.29	Online interview
16	ENGO staff	2022.03.29	Online interview

Third, as Table 4 reveals, we collected documents related to the Laohegou project. Two Chinese researchers completed the collection of these textual data. The data sources were the LNCC, the Paradise Foundation, and the Pingwu County Forestry Bureau. These archival materials were essential in understanding the operational processes of the project and its practical outcomes.

**Table 4:** Document data

Source	Content
The government	Agreement between the Pingwu County government and the Foundation, approval from the Pingwu County government for the establishment of the Laohegou Nature Reserve, approval from the Sichuan Provincial Forestry Department, and approval from the Mianyang Forestry Bureau
The foundation and LNCC	Laohegou Protected Area Management Manual, Baseline Survey, Paradise Foundation (Sichuan) Annual Report (2017–2019), Paradise Foundation (Sichuan) Financial Status (2017–2019), Paradise Foundation (Shenzhen) Annual Report (2016–2020)

## Case context

The Chinese government initiated collective forestry rights reform in 2008, thus allowing for the transfer and renting of forest land management rights. It created opportunities for ENGOs to manage land, known as land trusts. In 2010, ENGO-T persuaded the State Forestry Administration and the Sichuan Forestry Department to establish a land trust reserve pilot program. The following year, ENGO-T helped establish the Sichuan Nature Conservation Foundation (SNCF), which is funded by 22 Chinese entrepreneurs. ENGO-T provided technical support to the Foundation. In 2015, several employees left ENGO-T to join the SNCF and changed its name to the Paradise Foundation.^1^ Since then, the Foundation has operated the Laohegou project independently.

Pingwu County (32.42° N, 104.53° E) was selected for the pilot project for four main reasons. Firstly, it holds significant ecological importance as the giant panda is a species of great concern to both the international community and the Chinese government. Located in a priority area for biodiversity conservation, Pingwu County serves as the home to more than one-sixth of China’s giant panda population.^2^ Secondly, the project leveraged the existing connection between ENGO-T and the Sichuan Provincial Government, as they had previously collaborated on forestry carbon sink initiatives before the Laohegou project. Thirdly, Pingwu County has a tradition of cooperating with international ENGOs, like World Wildlife Foundation, in giant panda conservation efforts dating back to the 1980s. Lastly, the presence of conservation gaps in the county played a role in the selection. While three government-managed nature reserves are present, they cover only 37% of the pandas’ suitable habitat (Chen 2009). Consequently, in 2012, the Foundation launched the land trust nature reserve program in Pingwu County, with Laohegou as the chosen site. Laohegou locates along the biological corridor of two national nature reserves and a state-owned forestry farm. Despite the logging cessation after 1998, this region still faced human activity challenges such as hunting, fishing, and collecting medicinal herbs.

**Fig. 3:**
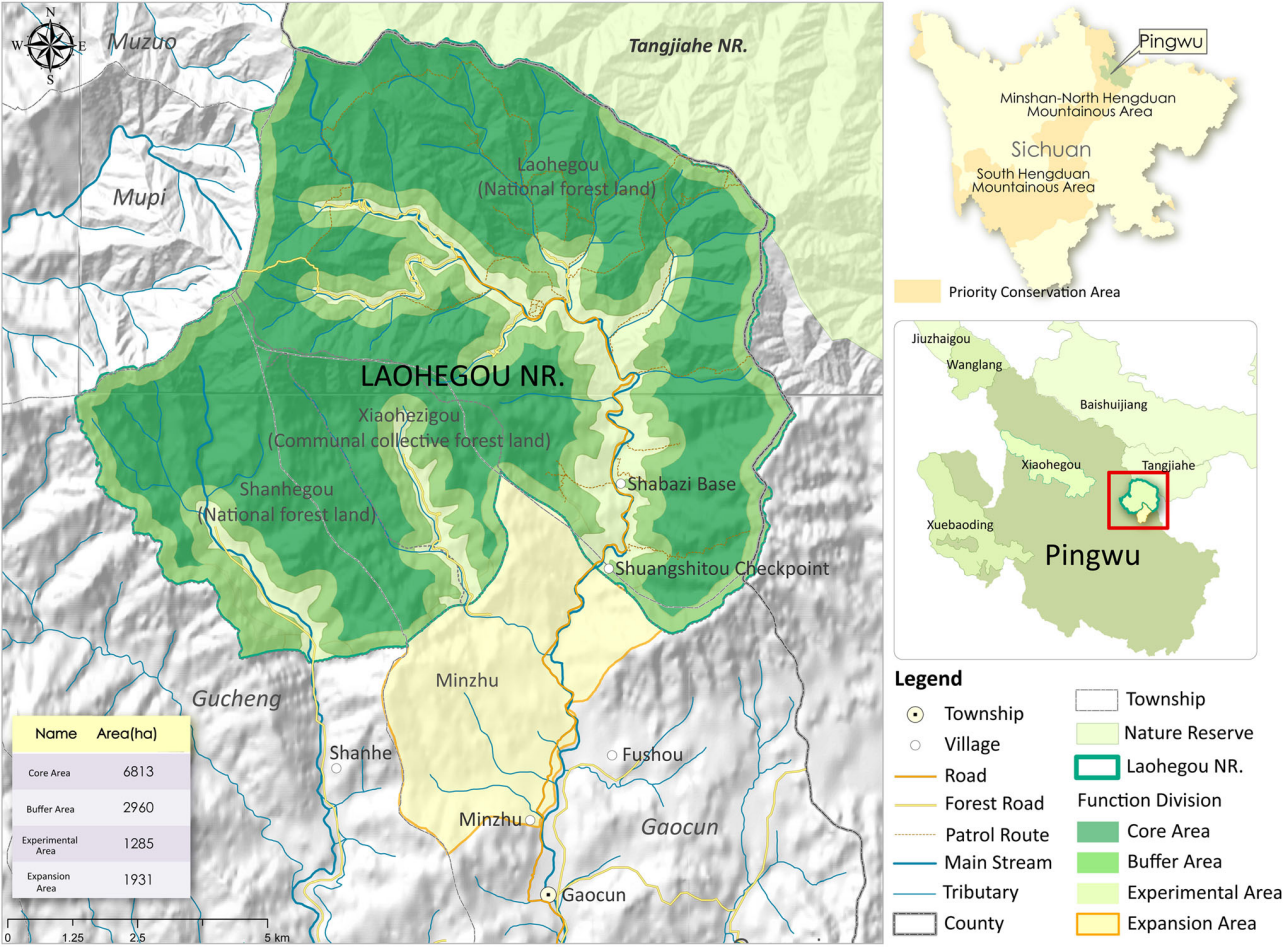
Location of Laohegou Nature Reserve (This figure does not cover protected areas that were newly added in 2020). *Source* Data from ENGO-T

In 2012, the Pingwu County government signed a land conservation agreement with SNCF. Under the agreement, the government leased 99 square kilometers of state-owned forest, of which 72 square kilometers belonged to Laohegou Forestry Farm, to the Foundation for 50 years at no cost. In the same year, the government of Gaocun Township transferred 11 square kilometers of collective forest to SNCF for 46 years for a fee of RMB 525 per ha. The agreements stipulate that the Foundation is responsible for wildlife and forest conservation in the area under the supervision of the Sichuan Forestry Department and the Pingwu County Forestry Bureau. As shown in Fig. 3, in 2013, the Pingwu County government approved the establishment of Laohegou as a county-level nature reserve, which was the first nature reserve in China to be managed by an ENGO.

The LNCC was registered with the Pingwu County government in 2014. This local organization is funded and managed by the Foundation, with supervision and technical guidance from the Pingwu County Forestry Bureau. The LNCC is responsible for the daily operations of the Laohegou Nature Reserve. Specifically, LNCC employees control entry to the reserve, conduct poaching patrols, collect monitoring data, and help surrounding communities develop eco-friendly agriculture. In 2020, the Giant Panda National Park Authority entrusted an additional 43 square kilometers of protected area to the LNCC, thus expanding its total area to 153 square kilometers.

## Results

### Evaluation of the Laohegou project

Considering factors such as project objectives, disciplinary background, and data access, evaluating the effectiveness of PPP project can be challenging (Hodge 2010). We, therefore, looked to the definition of PPP to guide our evaluation. Van Ham and Koppenjan (2001, p. 598) defined a PPP as a “cooperation of some durability between public and private actors in which they jointly develop products and services.” We extracted three indicators from this definition to evaluate the Laohegou project: common purpose, durability, and resource sharing.

Our evidence suggests that the Laohegou project has been aligned with the definition of the PPP. There are three main reasons. First, the Laohegou project has increased the total protected area in Pingwu County and improved connectivity between reserves. Moreover, a more varied range of stakeholders (e.g., corporations, communities, universities) have been able to participate in the Laohegou project. These achievements meet the expectations of PPAs (Holmes 2013; Cortés Capano et al. 2019). Second, the Laohegou project has been operating for 10 years now. Its long-term operation can be attributed to not only the explicit delineation of roles between the government and ENGOs but also the ENGOs’ ability to manage reserves^3^ and finances,^4^ which are considered crucial challenges for PPAs (Pasquini et al. 2011; Stolton et al. 2014; Hora et al. 2018). Third, the government and the ENGOs have collectively provided complementary resources for the Laohegou project (elaborated below in Section "Balance of Power and Interests between the Government and ENGOs at the Informal Dimension").

The fact that the Laohegou project meets the basic requirements of PPP does not mean, however, that highly coordinated relationships have developed between the various actors. Interorganizational balance requires ideological consensus (Benson 1975). In this regard, there is little agreement between the government and ENGOs regarding conservation strategy, especially with regard to tourism.^5^ Based on the balance of power, there is still friction between actors in project operations.^6^ Nevertheless, the two sides are not caught in a zero-sum game. Rather, they have achieved some degree of interest in interaction and been relatively satisfied, which underpins more collaboration. Starting in 2017, ENGOs and the government promoted the construction of a network of community nature reserves around the Laohegou Nature Reserve. Thus far, two villages have become involved.^7^ The same year, the first cross-jurisdictional joint patrol was established. The participants were LNCC, the Forestry Bureau, local communities, and state-owned nature reserves.^8^ In conclusion, although there is still friction between actors in the project’s operations, the basic PPP requirements have been fulfilled and the possibilities of innovative management have emerged. Thus, our overall evaluation of the Laohegou project remains positive.

### Partnership between the government and ENGOs at the formal dimension

The shared objectives of the government and ENGOs regarding biodiversity conservation are the starting point for cooperation. Furthermore, both parties have also invested resources such as coercion, financial incentives, and expert knowledge, respectively, for formal objectives, which has supported the operation of the Laohegou project.

#### Formal objectives

The government and ENGOs share the same formal objective in nature conservation. As the jurisdiction is a precious habitat for giant pandas, the Pingwu County government places high priority on wildlife conservation. Pingwu County’s first nature reserve was established in 1965, which was followed by two more reserves. Since 1998, all forestry has been halted in Pingwu County, and the forestry sector has shifted toward forest protection. Hence, forest and biodiversity protection has become a major political task for the local government (Pingwu County Government 2022). ENGO-T, which is one of the leading ENGOs worldwide, entered China in 1998 to promote the establishment of national parks in Yunnan Province. Meanwhile, the Foundation is also active in Sichuan, Jilin, and Anhui Provinces, with a focus on biodiversity conservation.^9^

Another common goal of the government and ENGOs is to support community development. This process includes purchasing rural residents’ agricultural products (e.g., walnuts, soybeans, Chinese herbs), providing funds to promote community-based nature conservation, and providing education funds for village children. Supporting community development was a government requirement when the Laohegou project was first established. The ENGOs are also aware of the ways in which local communities disturb wildlife.^10^ Therefore, they have made it their objective to find alternative livelihoods for local communities. In 2017, they partnered with Ant Forest, a public interest-oriented company, to promote community nature reserves.

#### Complementarity between the government and ENGOs in their partnership

The relative advantages of the government and ENGOs form the basis of their cooperation. Both have provided different resources to the Laohegou Nature Reserve to ensure the establishment and operation of the reserve.

1. The government’s coercive support

Importantly, the government has applied coercive resources to the reserve. The government confers legitimacy on the reserve and the LNCC, thus forming the basis for the PPP project. Legitimacy is transmitted top-down in a hierarchical system. Prior to the county government’s formal approval, the Sichuan Province Forestry Department and Mianyang Municipal Forestry Bureau approved the land trust reserve pilot project in 2011. It ensured the legitimacy of the Laohegou Nature Reserve at the legal and political levels.

A case demonstrates the government’s support for the Laohegou Nature Reserve. In 2016, the LNCC’s infrared cameras captured two men carrying guns. The information was passed to the government, and the two men were apprehended by police the following year. In August of that year, the county court established a circuit court in Laohegou and sentenced one man to a year and the other to nine months in prison for illegally carrying firearms.^11^

In addition, the county government granted the Laohegou Nature Reserve the right to enforce the law. This move overcame the ENGO-T’ difficulty of not having enforcement authority, which plagues many private actors (Hatchwell 2014; Stolton et al. 2014). Eight LNCC employees were assigned as forest police under the direction of the Forestry Public Security Bureau. They are equipped with police uniforms and have the authority to stop poaching and herb harvesting.

(2) Financial resources from ENGOs

While the government provides legitimacy and enforcement authority, the Foundation offers financial and information support to the nature reserve. With endorsements from entrepreneurs, the Foundation committed to raising RMB 180 million between 2012 and 2015, thus helping to give the Laohegou Nature Reserve financial security.^12^ Between 2017 and 2019, the Foundation invested RMB 8.63 million in the reserve.^13^ In addition to direct investment, in 2015, the Foundation helped establish a social enterprise to financially contribute to the reserve through the sale of honey products.^14^ The Foundation’s investments have not only guaranteed LNCC employee salaries, their living expenses, and the purchase of equipment but also supported the development of eco-friendly agriculture in the surrounding communities. By 2020, the Foundation had helped rural residents accumulate an additional income of RMB 1.5 million through purchasing agricultural products.^15^ These alternative livelihoods have reduced the reliance of peasants on hunting and herb gathering.

These financial resources also provide coercive power for ENGOs is. A security room and fence has been installed at the entrance to the reserve, and vehicle access requires application and clearance. Interestingly, infrared cameras also play a coercive role. As described in the case above, ENGOs can check for outsiders based on the photos from the infrared cameras. It gives a certain deterrent to peasants in the surrounding communities.^16^

(3) Expert knowledge of ENGOs

The Foundation began with conservation action planning for the Laohegou project. Specifically, it identified eight conservation targets, including giant pandas and lowland secondary broadleaf forests, through literature review, expert consultation, and participatory workshops. Simultaneously, the Foundation identified the need to address the lack of legal protection and management resources, and the demands of nearby communities for economic benefits. These steps mitigated threats such as illegal hunting and fishing.^17^

Subsequently, the Foundation invited research institutions such as the Chinese Academy of Sciences and Peking University to conduct a two-year survey of the state of flora and fauna in Laohegou. Through the baseline survey, researchers not only discovered endangered species such as the Asian golden cat but also found that the density of wild pandas in Laohegou was four times higher than that in the neighboring national nature reserve.^18^ Ultimately, these data underpinned the official approval of the Laohegou Forest Area as a county-level nature reserve.

Accordingly, the Foundation also developed a relatively simple and reliable ecological monitoring program. With the Foundation’s training, the LNCC staff acquired skills such as infrared camera operation,^19^ species identification, equipment uses (e.g., GPS, binoculars), and first aid. In addition to expert knowledge, the Foundation drew on the indigenous knowledge of local forestry staff to develop a patrol route for the reserve.^20^

### Balance of power and interests between the government and ENGOs at the informal dimension

Both formal and informal interests are the driving force behind actors’ engagement in the project. As both parties are mutually dependent on each other for the project’s operation, a balance of power is achieved, and certain informal interests are guaranteed for both parties. The informal dimension of the interaction between the government and ENGOs supports the cooperation between the actors and enables the project to function effectively.

The experience of the Pingwu County government and ENGOs in PPPs also allows for gaming and negotiation, which enhances the resilience of their cooperation. ENGO-T, in particular, has not only been involved in promoting nature conservation projects in Chinese Yunnan Province but also possesses prior experience in implementing land trusts in other countries. As a result, it comes into the Laohegou project with some expectation of potential challenges and the need for effective communication. On the side of public actors, the Pingwu County government has a long-standing history of engagement with ENGOs since the 1980s and has received support and endorsement from higher levels of government. This history of collaboration and the establishment of trust between the public and private actors serve as the foundation for ongoing interaction and consultation between the two sides.

#### Informal interests behind the formal objectives

Although the Laohegou project meets the formal objectives of the government and ENGOs, there are still potential conflicts of informal interests. Despite these interests are not the public statements of the government and ENGOs, we can find their pursuit of interests in their actions and test their benefits in the project effects. These empirical evidences display political, economic, and strategic self-interest in the operation of the Laohegou project.

First, it is a primate challenge for ENGOs to gain the trust of the government while maintaining their independence (Song et al. 2015; Yuen 2018). Local governments in China are responsible for everything in their territories, which makes them try to ensure their control (Zhou 2017). To this end, ENGO-T promotes the establishment of the Foundation and the LNCC, both of which are regulated by the government.^21^ Meanwhile, ENGOs strive to keep dominance over the program.^22^

Second, organizations would seek to get more budget for themselves (Peters 2018). The ban on natural forest logging, implemented in 1998, has curbed the Pingwu County government’s fiscal interest for forestry logging (Zhao et al. 2023). However, the local government continues to hope for more funding from ENGOs, and expects the project to not only fund conservation in the region but also lead to grants for forestry employees and the community. Meanwhile, ENGOs are also under financial pressure and hope the government will transfer funds to them for the Natural Forest Protection Project.

Third, there are some differences in the nature conservation strategies from government and ENGOs, although they share common goals for wildlife conservation. The government wants to promote tourism whenever possible while protecting forests and endangered wildlife (e.g., the giant panda). The ENGOs, however, want to reduce human activity throughout the Laohegou region, including total bans on hunting, fishing, and herb harvesting, and allowing a limited range of nature education activities. They also want to reduce the dependence of communities on the ecosystem through alternative livelihoods.

#### Actors’ benefits in the balance of power

At both formal and informal levels, the power elements of the actors play a dual role. On the one hand, the power resources of the government and ENGOs support the operation of the Laohegou project. On the other hand, both sides may use power elements to achieve informal interests. However, in the Laohegou project, both sides rely on each other's resource input, leading to a status of power balance (Avelino and Wittmayer 2016).With this balance of power, the government and ENGOs pursue certain informal interests while also considering the concerns of the other party, who can threaten to diminish resources to protect their interests. Each side seeks to satisfy its own interests within the other side’s acceptable limits, which underpins the successful implementation of the Laohegou project.

1. Balance of control over the project

The control of the Laohegou project is a matter of utmost negotiation between the two parties. Right from the project's inception, the government and ENGOs have included the division of management rights in the contract. ENGOs were granted exclusive management rights to the land for a period of 50 years. Meanwhile, the Pingwu County Forestry Bureau agreed to become the sponsoring institution for the LNCC. It gave the Forestry Bureau not only the responsibility for supervising the LNCC but also the legal status and professional knowledge needed to control it. Through the cooperation agreement, the government retains the ability to take coercive action against the Foundation and the LNCC. If the Foundation fails to perform, the government can revoke ENGOs’ management rights or hold them liable for violations.

While the cooperation agreement is pivotal, it does not signify the conclusion of the power balance between the government and ENGOs. After the formalization of the Laohegou Project, the government and ENGOs further refine the control of both parties in the administrative process. The Foundation utilizes its economic and informational powers to ensure autonomy in nature reserve management. The Foundation appoints LNCC personnel and provides employee salaries, including the three directors of the LNCC. Aside from maintaining control of the LNCC in terms of personnel and finances, the Foundation’s staff in Chengdu frequently visit the Laohegou Nature Reserve to inspect operations, receive reports, and develop work plans.

Simultaneously, the government uses coercive resources to assert formal control over the nature reserve. The LNCC is required to report to the Forestry Bureau every two months on its activities, including conservation effectiveness and species monitoring data. This mechanism allows the government to maintain oversight and exert influence over the project’s progress.

(2) Mutual reduction of economic claims

ENGOs and the Pingwu County Government engaged in a complex arrangement regarding the staffing and funding of the LNCC. At the request of the government, ENGOs hired 17 workers from the state-owned forestry farm to work at the LNCC. However, these employees were reluctant to give up their subsidies from the Natural Forest Protection Project, resulting in them signing labor contracts with ENGOs but not transferring their official positions.

This situation remained dynamic and subject to consultation and negotiation. The forestry workers lacked effective motivation, leading to tensions between ENGOs and the forestry farm. At one point, the foundation's leaders even contemplated terminating the Laohegou project due to these challenges. However, the Pingwu County government did not want to terminate the cooperation based on this issue. Through negotiation, both parties made concessions on their economic claims.

Ultimately, in 2020, the Foundation ceased paying the salaries of the forestry employees, and the government transferred them to work in other forest areas. In exchange, the Foundation refrained from seeking funding of the Natural Forest Protection Project. This compromise served as a resolution to the staffing and funding complexities between the ENGOs and the Pingwu County Government concerning the LNCC.

(3) Strategies under continuous consultation

In terms of strategic interests, the strategies of the public and private sectors are not entirely compatible. Some of the Foundation’s tactics such as community-based nature conservation does not contradict the government’s interests and are, therefore, supported by the latter. With the government’s endorsement, the LNCC began promoting community-based conservation plots in 2017.

By contrast, the government and the Foundation compete with regard to tourism objectives. The Foundation wants to strictly control the effects of human activity on the reserve and only allows certain nature education activities. The government, meanwhile, has continued to expect tourism development in the Laohegou area and has cooperated with certain companies in this regard. However, with the persistent efforts of the LNCC and the Foundation, tourism development plans were shelved for the time being. Thus, in this type of game, the two parties had identified each other’s objectives—developing tourism and limiting human activity in the area. On that basis, the Foundation and the government have continued to negotiate tourism proposals.^23^

## Discussion

### PPPs beyond polity differences: A dialog with the authoritative environmentalist literature

In the realm of nature conservation, discussions on PPPs have centered on Western democracies like the United States and Australia (Stolton et al. 2014; Fitzsimons 2015; Runhaar et al. 2017), or countries with prevalent neoliberal ideologies, such as Chile (Holmes 2015). However, the case study of the Laohegou project reveals that PPPs can also prove effective as a governance strategy in an authoritarian polity. Through interdependent cooperation, such partnerships can transcend political differences and become a universal solution (Ansell and Gash 2018). By ensuring relative independence for both the government and ENGOs, they can establish a formal platform to collaboratively set goals, share resources, and shoulder responsibilities (Ansell and Gash 2007, 2018).

Existing research on authoritarian environmentalism has often depicted society as resisting and the state as suppressing (Beeson 2010; Li and Shapiro 2020). However, the model of cooperation showcased in the Laohegou project demonstrates the potential for the Chinese government and ENGOs to coexist and work together based on their respective interests, fostering collaboration rather than confrontation (Song et al. 2015). This refutes the notion of a zero-sum game between the government and society. As biodiversity faces alarming decline, the Chinese government has shown an increasing inclination toward engaging with ENGOs, while the ENGOs themselves are not solely critics but also willing to collaborate with the government (Xu and Byrne 2021; Zhao et al. 2023).

Similar to PPPs in other countries, China's practice highlights the significance of decentralization and platform mechanisms (Ansell et al. 2022). However, China encounters various challenges. Firstly, the country's decentralization mechanism in environmental governance has undergone significant changes under Xi Jinping’s leadership (Qiaoan and Teets 2020; Hsu et al. 2021). While the top leader's focus on environmental issues has led to shifts in local government priorities, the upward transfer of decision-making power from the central government may restrict the space for local actors to pursue policy innovations (Zhao et al. 2023). Secondly, the platform mechanism between public and private actors in China appears to be less stable. Cooperation relies on informal consultations, individual contacts, endorsements from other official institutions, and trust accumulated through multiple rounds of interaction (Yuen 2018; Yang and Zhang 2021). As such, China still needs to explore suitable political and social elements to further support public–private cooperation.

### Power balance directing more collaborations: Contributions to land trusts research

This study confirms that cooperative relationships in land trusts are fundamentally shaped by the interests and power dynamics among actors (Benson 1975; Sack 2019). Economic interests of landowners, for instance, can either facilitate the establishment of a land trust program or jeopardize its long-term sustainability. Actors often harbor informal interests that go beyond formal objectives, and power plays a critical role in safeguarding these interests (Krott et al. 2014; Rahman and Giessen 2017). In the context of Chinese studies, scholars have expressed concerns about government control undermining NGOs’ autonomy (Ho 2001; Spires 2011; Yang and Zhang 2021). Conversely, other researchers argue that overly powerful NGOs can suppress government involvement (Burns et al. 2017). Thus, maintaining a cooperative relationship necessitates a power balance for the involved actors, while resource investments in PPPs serve as guarantees for realizing their interests in the informal dimension.

This study adopts a political science perspective that deviates from adversarial politics, which assumes a winner-takes-all approach (Busenberg 1999; Ansell and Gash 2007). Instead, it highlights that while antagonistic relations between actors may be unavoidable, common goals can steer them from confrontation toward more cooperative actions (Benson 1975; Ansell and Gash 2007). A balance of power allows actors to make cumulative progress toward cooperation, even amid persistent frictions (Ansell and Gash 2007).

Achieving the balance of power and interests is not a quick process but requires a long-term approach to reach a relatively satisfactory state for both parties. Based on the case study, two key points emerge. Firstly, PPP mechanisms like land trusts necessitate the establishment of an institution for long-term consultation. This can manifest through informal interactions similar to the Laohegou project or be formalized within the terms of the land trust contract (Cheever 1996). Secondly, both parties could build trust through incremental “small wins.” Trust accumulation is crucial for the long-term game, and mutual confirmation of formal objectives and boundaries is achieved through multiple rounds of communication. In the Laohegou project, the parties fostered trust and increased commitment and resources through joint patrols and community conservancies.

## Conclusion

Based on a political science perspective, we proposed that shared advantages between actors are a necessary but not sufficient condition for PPPs. In interorganizational networks, governments and ENGOs not only exploit their advantages to achieve formal goals but also utilize power instruments to pursue informal self-interest. Thus, forming a partnership also requires a balance of power and interests between actors. The case of the Laohegou project provided empirical evidence for our two working hypotheses. Working hypothesis 1 proposes that government coercion and monetary and informational resources from ENGOs produce the innovative management of the Laohegou Nature Reserve through PPP. Working hypothesis 2 proposes that the balance of power and interests between the government and ENGOs determines support for Laohegou PPP management. The power resources of the government and ENGOs form the basis for the operation of the Laohegou project. Moreover, the government and the ENGOs do not have sufficient power to suppress each other. They cannot, therefore, ignore each other when pursuing political, economic, and strategic interests. The balance of power and interests between the government and the ENGOs facilitates the project’s operations.

This study used a political science perspective to analyze the relationship between government and ENGOs and develop the concept of political embeddedness. As an exploratory case study, however, it was not strictly possible to test the hypotheses. Instead, we presented empirical evidence for two working hypotheses. Constrained by a single case, this study has inherent limitations in generalizability. Further research is therefore necessary. The comparative research with a stronger empirical basis is needed to test the hypotheses in the Chinese context. Moreover, cross-country comparisons could examine the similarities and differences between cases in China and other countries from a political science perspective. Ultimately, we sought to demonstrate the scientific usefulness of our analytical lens.

### Supplementary Information

Below is the link to the electronic supplementary material.

Supplementary file1 (PDF 323 kb)

## Footnotes

1 To avoid confusion, we use “the Foundation” to collectively refer to the SNCF and the Paradise Foundation.

2 Sichuan Provincial Forestry Department, 2015. The 4th Survey Report on Giant Pandas in Sichuan Province. Sichuan Science and Technology Press, Chengdu (China).

3 Interviews 8 and 9. Many of the ENGO members involved in the Laohegou project have experience with other nature conservation projects.

4 Interview 3. On the one hand, the Foundation has strong financial management capability, which is reflected in the annual report. On the other hand, the LNCC is staffed by professional financial management personnel.

5 Interview 16.

6 Interviews 15 and 16.

7 Interviews 10 and 16.

8 Observation by the researcher in 2017.

9 Paradise Foundation (Shenzhen) Annual Report (2016–2020).

10 Gathering medicinal herbs and hunting are important means of livelihood for local residents. With their forest land adjacent to the reserve, they can easily move over the mountains to the Laohegou area. When the project first began, ENGO staff recalled hearing gunshots in the region (Interview 8).

11 Interview 2.

12 Agreement between the Pingwu County government and the Foundation.

13 Paradise Foundation (Sichuan) Financial Status (2017–2019).

14 In 2015, the Foundation established a social enterprise, Laohegou Baihua Valley Honey Co. In 2017, the Foundation invested an additional RMB 2.4 million in this corporation.

15 Paradise Foundation (Shenzhen) Annual Report (2016–2020).

16 Interview 11.

17 Interview 8.

18 Interview 9.

19 In addition to collecting wildlife data, infrared cameras have become an important tool for deterring local residents from entering (Interviews 2 and 11).

20 According to observations, many patrol paths in the Laohegou Nature Reserve originated from former forestry logging operation routes.

21 Interview 8.

22 Interview 16.

23 Based on interview 15, a possible scenario would be to accommodate tourists in the surrounding communities rather than in the reserve and give them access to the reserve for visits.
