# Supplementary material for: Political embeddedness in public–private partnership for nature conservation: A land trust reserve case from China
Source: Ambio. 2023 Oct 11;53(2):324–38. doi: 10.1007/s13280-023-01936-y (PMC10774467; doi:10.1007/s13280-023-01936-y)
Supplement: Supplementary file 1 — Supplementary file1 (PDF 323 kb) [file 13280_2023_1936_MOESM1_ESM.pdf]

Ambio

Electronic Supplementary Material

This supplementary material has not been peer reviewed.

Title: **Political Embeddedness in Public–Private Partnership for Nature Conservation: A Case Study from China**

Authors: Jiacheng Zhao, Tong Jin, Pei Zhang, Max Krott, Jinlong Liu

APPENDIX A: SAMPLE INTERVIEW QUESTIONS

1. Process of the Laohegou Project Establishment

A. Why was the land trust mechanism chosen in 2012?

B. Why was the Laohegou area in Pingwu County, Sichuan Province chosen as the project site?

C. How was the ENGO team organized at that time?

D. What government administration was involved at that time?

E. What was research conducted before the Laohegou project was formalized?

F. What were the key matters of the land trust contract? How did the two parties reach a consensus?

2. Key Details of the Laohegou Project Implementation Process

A. How was the Laohegou Project funded?

B. How were LNCC workers recruited?

C. How did the ENGOs' personnel work with LNCC staff? How was the content of LNCC's task set? How is it supervised?

D. How does LNCC communicate with the Pingwu County government?

E. How does the Laohegou project achieve cooperation with the community?

3. Challenges

A. What do you think are the most significant challenges of the Laohegou Project?

B. How has the government/ENGOs responded to these challenges?

C. How do you think these issues have affected your relationship with the government/ENGOs?

4. Other

A. Has the current status of the Laohegou project met your initial expectations?

B. What are your expectations for the future of the Old River Gorge Project?
